# Determinants of saxagliptin use among patients with type 2 diabetes mellitus treated with oral anti-diabetic drugs

**DOI:** 10.1186/s40360-015-0007-z

**Published:** 2015-04-02

**Authors:** M Elle Saine, Dean M Carbonari, Craig W Newcomb, Melissa S Nezamzadeh, Kevin Haynes, Jason A Roy, Serena Cardillo, Sean Hennessy, Crystal N Holick, Daina B Esposito, Arlene M Gallagher, Harshvinder Bhullar, Brian L Strom, Vincent Lo Re

**Affiliations:** 1 Department of Biostatistics and Epidemiology, Center for Clinical Epidemiology and Biostatistics, Perelman School of Medicine at the University of Pennsylvania, 423 Guardian Drive, Philadelphia, PA USA; 2 Department of Biostatistics and Epidemiology, Center for Pharmacoepidemiology Research and Training, Perelman School of Medicine at the University of Pennsylvania, Philadelphia, PA USA; 3 HealthCore, Inc, Wilmington, DE USA; 4 Department of Medicine, Perelman School of Medicine at the University of Pennsylvania, Philadelphia, PA USA; 5 Clinical Practice Research Datalink, Medicines and Healthcare Products Regulatory Agency, London, UK; 6 Cegedim Strategic Data Medical Research, London, UK; 7 Rutgers Biomedical & Health Sciences, Rutgers, the State University of New Jersey, Newark, NJ USA

**Keywords:** Diabetes mellitus, Saxagliptin, Dipeptidyl peptidase IV inhibitor, Pharmacoepidemiology

## Abstract

**Background:**

The patterns and determinants of saxagliptin use among patients with type 2 diabetes mellitus (T2DM) are unknown in real-world settings. We compared the characteristics of T2DM patients who were new initiators of saxagliptin to those who were new initiators of non-dipeptidyl peptidase-4 (DPP-4) inhibitor oral anti-diabetic drugs (OADs) and identified factors associated with saxagliptin use.

**Methods:**

We conducted a cross-sectional study within the Clinical Practice Research Datalink (CPRD), The Health Improvement Network (THIN), US Medicare, and the HealthCore Integrated Research Database (HIRD^SM^) across the first 36 months of saxagliptin availability (29 months for US Medicare). Patients were included if they were: 1) ≥18 years old, 2) newly prescribed saxagliptin or a non-DPP-4 inhibitor OAD, and 3) enrolled in their respective database for 180 days. For each saxagliptin initiator, we randomly selected up to ten non-DPP-4 inhibitor OAD initiators matched on age, sex, and geographic region. Conditional logistic regression was used to identify determinants of saxagliptin use.

**Results:**

We identified 64,079 saxagliptin initiators (CPRD: 1,962; THIN: 2,084; US Medicare: 51,976; HIRD^SM^: 8,057) and 610,660 non-DPP-4 inhibitor OAD initiators (CPRD: 19,484; THIN: 19,936; US Medicare: 493,432; HIRD^SM^: 77,808). Across all four data sources, prior OAD use, hypertension, and hyperlipidemia were associated with saxagliptin use. Saxagliptin initiation was also associated with hemoglobin A1c results >8% within the UK data sources, and a greater number of hemoglobin A1c measurements in the US data sources.

**Conclusions:**

In these UK and US data sources, initiation of saxagliptin was associated with prior poor glycemic control, prior OAD use, and diagnoses of hypertension and hyperlipidemia.

**Trial registration:**

ClinicalTrials.gov identifiers NCT01086280, NCT01086293, NCT01086319, NCT01086306, and NCT01377935

## Background

Type 2 diabetes mellitus (T2DM) is a global public health problem, affecting 347 million people worldwide [1,2]. Current estimates suggest that more than 29.1 million adults and children in the United States (US) and 3.2 million adults and children in the United Kingdom (UK) have T2DM, representing more than 9.3% and 6% of these populations, respectively [3-5]. Oral anti-diabetic drugs (OADs), along with diet and exercise, can help to control T2DM-associated hyperglycemia in adults [6].

Saxagliptin, a relatively new dipeptidyl peptidase-4 (DPP-4) inhibitor [7], was approved by the US Food and Drug Administration (FDA) in July 2009 and the European Medicines Agency in October 2009 to be used with diet and exercise to control hyperglycemia in adults with T2DM. In clinical trials, saxagliptin was shown to be efficacious in lowering fasting plasma glucose, 2-hour postprandial glucose, and hemoglobin A1c when used as monotherapy [8], in combination with metformin in treatment-naive patients [9], or as add-on therapy to metformin [10], sulfonylureas [11], thiazolidinediones [12], or insulin [13]. Because of its recent market introduction, prescribing patterns associated with saxagliptin’s use in real-world settings remain unknown. Determining how OADs are prescribed in clinical practice can provide valuable information on healthcare decision-making [14,15]. Further, since the effectiveness and safety of saxagliptin and other OAD therapies may be affected by demographic characteristics, medical comorbidities, and additional medications prescribed to T2DM patients, identifying the factors associated with the use of particular OADs in real-world settings can provide important information needed for the future conduct of studies evaluating the comparative effectiveness and safety of anti-diabetic drugs. In particular, such variables can be incorporated within propensity scores to help to minimize confounding by indication [16,17].

The objectives of this study were to: 1) compare the characteristics of patients with T2DM who newly initiate saxagliptin to those who newly initiate OADs in classes other than DPP-4 inhibitors, and 2) identify determinants of saxagliptin use during the first years of its availability in the UK and US. We hypothesized that T2DM patients with poor glycemic control, a higher prevalence of microvascular and macrovascular complications, and prior OAD use would be more likely to initiate saxagliptin.

## Methods

### Data sources

Four data sources, two each in the UK and US, were used in this study. Within the UK, data from the Clinical Practice Research Datalink (CPRD; formerly General Practice Research Database) and The Health Improvement Network (THIN) were evaluated over the first 36 months of saxagliptin availability (5 October 2009 to 30 September 2012). Within the US, data from US Medicare were evaluated across all 50 states over the first 29 months of saxagliptin availability (1 August 2009 to 31 December 2011), due to the lag in availability of these data. The HealthCore Integrated Research Database (HIRD^SM^) data were examined over the first 36 months of saxagliptin availability (1 August 2009 to 31 July 2012). These four databases were selected because they include large numbers of T2DM patients across all age groups and utilize health records (UK) and claims data (US) from both private and public insurance plans, providing broadly representative study samples.

Details on the data available within each of these data sources for the purpose of evaluating OAD use have been previously described [18]. At the time of data collection, CPRD contains electronic medical records of over 15 million UK patients across 684 practices [19], and THIN contained primary medical records for over 11 million UK patients across over 550 practices [20,21]. CPRD and THIN collect demographic information, medical diagnoses and surgical procedures (recorded using Read codes), outpatient laboratory results, and general practitioner-issued prescriptions [22,23]. Since some UK practices contribute data to both CPRD and THIN [24], we excluded overlapping patients from THIN data to ensure that these patients were not counted twice. US Medicare is the largest national health insurance program administered by the US federal government, serving approximately 47.5 million people as of 2010 [25]. Medicare is available to US citizens aged 65 years or older and those under 65 years with certain disabilities. The HIRD^SM^ is one of the largest longitudinal commercial health insurance databases in the US, serving 23.2 million members as of 2010 [26-28]. Both Medicare and the HIRD^SM^ contain demographic information, inpatient and outpatient medical diagnoses (recorded using International Classification of Diseases, Ninth Revision, Clinical Modification diagnosis codes), surgical procedures (recorded with Current Procedural Terminology codes), and dispensed medications (recorded by National Drug Codes). Although codes for ordered laboratory tests can be identified within Medicare and the HIRD^SM^, the results of these tests are not recorded in Medicare and are only available in a subset of HIRD^SM^ patients. To avoid the possibility of double-counting patients concurrently enrolled in both of these US data sources, we only included HIRD^SM^ data for persons aged 18–64 years and censored HIRD^SM^ enrollees at age 65 years.

The study was approved by the University of Pennsylvania and Rutgers University Institutional Review Boards, the Quorum Review Institutional Review Board (HIRD^SM^), and the Independent Scientific Advisory Committees for CPRD and THIN. A data use agreement was obtained from the Centers for Medicare and Medicaid Services (US Medicare).

### Study patients

Patients were eligible for study inclusion if they were: 1) newly prescribed (in the UK) or dispensed (in the US) either saxagliptin, as a single agent or in combination with other OADs, or an OAD in a class other than DPP-4 inhibitors (“index drug”); 2) ≥18 years old; and 3) enrolled in their respective data source for at least 180 days prior to initiation of their index drug. The rationale for selecting new initiators of other OADs as the comparator group was to study patients with diabetes who required initiation of new OAD therapy. Given that we wished to identify factors specifically associated with saxagliptin use, we did not include new initiators of other DPP-4 inhibitors within the comparator OAD group.

All eligible patients prescribed saxagliptin were included. Within each data source, a random sample (without replacement) of up to ten new initiators of non-DPP-4 inhibitor OADs was selected for each saxagliptin initiator. These patients were matched on age (within 5-year age groups), sex, and geographic region (i.e., country within UK data sources; census region within US data sources).

### Main study outcome

The main study outcome was a new prescription (UK data source) or pharmacy claim (US data source) for either saxagliptin or a non-DPP-4 inhibitor OAD. The index date was defined as the date of first prescription of saxagliptin or comparator OAD in the respective data source.

### Determinants of saxagliptin use

The following variables were evaluated as determinants of use of saxagliptin compared to other OADs: calendar year of initiation, medical comorbidities, surgical procedures, and medications of interest (listed in Table 1). In clinical practice, primary care physicians and endocrinologists likely select and prescribe oral anti-diabetic drugs based on consideration of at least many of these factors. We included a variety of medications and drug classes as potential determinants of saxagliptin because concerns for drug-drug interactions or exacerbation of medication toxicities might influence decisions to prescribe saxagliptin.

**Table 1:** **Demographic characteristics of type 2 diabetes mellitus patients within United Kingdom data sources**

	Clinical Practice Research Datalink			The Health Improvement Network		
Characteristic^*^	Saxagliptin	Other OAD	Standardized difference	Saxagliptin	Other OAD	Standardized difference
	(n = 1,962)	(n = 19,484)		(n = 2,084)	(n =19,936)	
**Mean (SD) age, years** ^†^	52.7 (10.6)	52.2 (10.6)	0.01	64.7 (12.9)	64.6 (12.9)	0.01
**Male sex**^**†**^	58.2%	58.2%	<0.01	57.7%	57.0%	0.01
**UK country**^†^						
England	60.4%	60.6%	<0.01	63.1%	65.7%	0.05
Northern Ireland	7.1%	7.0%	<0.01	5.3%	5.0%	0.01
Scotland	11.4%	11.5%	<0.01	12.3%	12.8%	0.01
Wales	21.0%	20.9%	<0.01	19.2%	16.5%	0.07
**Other OAD initiated at index date**						
Alpha-glucosidase inhibitors: Acarbose	0%	0.2%	-	0%	0.2%	-
Biguanide: Metformin	0%	63.1%	-	0%	62.1%	-
Meglitinides	0%	0.4%	-	0%	0.4%	-
Nateglinide	0%	0.1%	-	0%	0.0%	-
Repaglinide	0%	0.3%	-	0%	0.3%	-
Sulfonylureas	0%	27.9%	-	0%	29.1%	-
Glibenclamide (Glyburide in US data sources)	0%	0.2%	-	0%	0.2%	-
Gliclazide	0%	24.9%	-	0%	26.1%	-
Glimepiride	0%	2.1%	-	0%	1.9%	-
Glipizide	0%	0.6%	-	0%	0.7%	-
Tolbutamide	0%	0.1%	-	0%	0.2%	-
Thiazolidinediones	0%	8.3%	-	0%	8.2%	-
Pioglitazone	0%	8.1%	-	0%	8.0%	-
Rosiglitazone	0%	0.2%	-	0%	0.2%	-
**On glucagon-like peptide-1 receptor agonist**	3.1%	1.9%	0.08	2.4%	1.9%	0.03
**On insulin**	5.4%	7.3%	0.08	7.7%	7.2%	0.02
**Hemoglobin A1c measurements**						
Mean (SD)	8.7 (1.6)	8.6 (1.8)	0.06	8.8 (1.6)	8.6 (1.8)	0.08
Hemoglobin A1c >8%	57.6%	39.8%	0.36	56.2%	40.8%	0.31
**Mean body mass index (SD)**	32.4 (6.5)	31.5 (6.7)	0.14	-	-	-
Missing values	42.9%	41.4%	0.03	0.7%	2.0%	0.11
Underweight (15–18.5 kg/m^2^)	0.2%	0.4%	0.03	0.0%	0.4%	0.08
Normal (18.5-24.9 kg/m^2^)	4.4%	6.7%	0.10	7.9%	11.4%	0.12
Overweight (25.0-29.9 kg/m^2^)	15.9%	17.8%	0.05	29.0%	31.5%	0.05
Obese (30–60 kg/m^2^)	36.7%	33.9%	0.06	62.3%	54.7%	0.15
**Smoking**	36.5%	36.5%	<0.01	63.5%	61.0%	0.05
**Severity of type 2 diabetes mellitus (prior 180 d)**						
Cerebrovascular disease	0.5%	0.7%	0.02	0.9%	0.7%	0.02
Coronary artery disease, congestive heart failure, ventricular tachycardia/fibrillation	1.3%	1.5%	0.01	1.9%	1.5%	0.03
Diabetic coma	0%	0.1%	-	0%	0.1%	-
Nephropathy	0.3%	0.2%	0.02	0.6%	0.2%	0.06
Neuropathy	0.8%	0.6%	0.02	0.9%	0.6%	0.03
Peripheral vascular disease	1.1%	0.9%	0.02	1.2%	0.9%	0.03
Retinopathy	5.7%	3.7%	0.09	5.5%	3.5%	0.10
Unspecified additional diabetic complications	0%	0.0%	-	0%	0.0%	-
**Medical comorbidities**						
Allergic rhinitis/hay fever	8.8%	10.0%	0.04	9.1%	9.4%	0.01
Asthma	16.0%	15.8%	<0.01	16.4%	15.9%	0.01
Chronic obstructive pulmonary disease/bronchitis	12.1%	10.0%	0.07	13.1%	10.7%	0.07
Dermatologic disorder						
Eczema	17.0%	15.2%	0.05	16.9%	14.2%	0.08
Psoriasis/psoriatic arthritis	6.4%	5.1%	0.06	5.8%	5.3%	0.02
Gastrointestinal disease						
Cirrhosis	0.3%	0.4%	0.01	0.2%	0.4%	0.03
Gallbladder disease	6.2%	5.6%	0.02	6.3%	5.9%	0.02
Hemochromatosis	0.1%	0.1%	0.02	0%	0.2%	0.04
Hyperlipidemia	16.0%	12.0%	0.12	15.9%	12.2%	0.11
Hypertension	56.7%	50.9%	0.12	61.0%	56.1%	0.10
Infectious disease						
Hepatitis B virus infection	0.3%	0.2%	0.02	0.2%	0.2%	0.01
Hepatitis C virus infection	0%	0.1%	-	0%	0.1%	-
Malignancy						
Hematologic	0.8%	1.0%	0.03	0.9%	1.0%	<0.01
Solid organ	23.5%	22.9%	0.02	22.9%	23.9%	0.02
Obesity	17.8%	14.6%	0.09	17.0%	13.4%	0.10
Rheumatoid arthritis	2.7%	2.4%	0.02	1.3%	1.7%	0.03
**Medications**						
Acetaminophen/paracetamol	31.7%	28.7%	0.06	32.0%	30.1%	0.04
Anti-asthmatic agents	18.6%	17.7%	0.02	18.8%	18.1%	0.02
Antibacterials	35.5%	33.9%	0.03	32.1%	30.7%	0.03
Anticonvulsants	8.8%	7.8%	0.04	6.2%	5.1%	0.05
Antifungals	3.0%	2.6%	0.02	2.9%	3.4%	0.03
Antihistamines	7.2%	7.1%	<0.01	7.5%	6.9%	0.02
Anti-hyperlipidemic agents	80.6%	58.5%	0.49	81.1%	61.9%	0.43
Antihypertensive agents						
Angiotensin-converting enzyme inhibitors	46.5%	36.1%	0.21	46.1%	38.4%	0.16
Angiotensin receptor blockers	19.2%	13.2%	0.16	22.5%	15.0%	0.19
Beta blockers	24.7%	20.7%	0.09	28.0%	23.2%	0.11
Calcium channel blockers	28.2%	23.8%	0.10	29.9%	26.9%	0.07
Loop diuretics	12.4%	11.0%	0.04	17.3%	11.4%	0.17
Other antihypertensive agents	9.2%	6.8%	0.09	10.0%	6.8%	0.11
Thiazide diuretics	19.9%	16.1%	0.10	25.1%	20.8%	0.10
Antivirals	0.7%	0.8%	0.02	0.8%	0.9%	0.02
Non-aspirin non-steroidal anti-inflammatory	13.4%	13.0%	0.01	12.5%	13.0%	0.02
Other antiplatelet/anticoagulant agents						
Aspirin	38.3%	29.7%	0.18	42.1%	32.2%	0.21
Clopidogrel	4.9%	3.7%	0.06	5.0%	3.7%	0.06
Low-molecular-weight heparin	0.2%	0.3%	0.03	0.5%	0.3%	0.02
Warfarin	5.4%	4.9%	0.02	6.9%	5.6%	0.05
Other medications						
Allopurinol	3.2%	3.5%	0.02	5.1%	3.7%	0.07
Anti-arrhythmics	3.4%	2.6%	0.05	3.1%	3.0%	<0.01
Immune modulators/immunosuppressants	1.2%	1.2%	<0.01	1.2%	1.2%	0.01
Nitroglycerin	5.6%	5.1%	0.02	6.3%	4.9%	0.06
Urinary anti-spasmodics	4.3%	3.3%	0.05	4.7%	3.6%	0.06
Psychotropic agents						
Antidepressants	22.1%	19.6%	0.06	20.6%	19.6%	0.03
Antipsychotics	4.3%	4.4%	0.01	4.9%	4.8%	0.01
**Prior OAD Therapy** ^‡^	93.7%	35.8%	1.53	92.1%	36.8%	1.41
Alpha-glucosidase inhibitors: Acarbose	0.3%	0.1%	0.03	0.2%	0.1%	0.03
Biguanide: Metformin	83.4%	73.7%	0.24	77.7%	73.1%	0.11
Meglitinides	1.1%	0.4%	0.09	1.2%	0.3%	0.10
Nateglinide	0.2%	0.0%	0.04	0.2%	0.1%	0.04
Repaglinide	0.9%	0.3%	0.08	1.0%	0.2%	0.10
Sulfonylureas	47.9%	12.2%	0.84	46.4%	12.8%	0.79
Glibenclamide	0.4%	0.5%	0.01	0.5%	0.6%	0.01
Gliclazide	41.4%	12.6%	0.69	39.3%	13.4%	0.61
Glimepiride	4.5%	1.5%	0.18	4.1%	1.4%	0.17
Glipizide	1.1%	0.7%	0.04	1.7%	0.8%	0.09
Tolbutamide	0.7%	0.1%	0.09	1.1%	0.2%	0.11
Thiazolidinediones	16.6%	5.3%	0.37	17.7%	5.1%	0.40
Pioglitazone	14.1%	3.5%	0.38	15.4%	3.4%	0.42
Rosiglitazone	2.7%	2.1%	0.03	2.5%	2.0%	0.03

Abbreviations: OAD = oral anti-diabetic drug; SD = standard deviation.

^*^Characteristics are presented as percentages unless otherwise indicated.

^†^Matching criteria for which a random sample (without replacement) of up to ten new initiators of non-DPP-4 inhibitor OADs were selected for each saxagliptin initiator.

^‡^Defined as use of an oral anti-diabetic drug within the 180 days prior to the initiation of the index drug. Denominator adjusted to exclude those on index drug.

Comorbidities were identified based on the presence of diagnoses recorded in the 180 days prior to the index date within US Medicare and HIRD^SM^, and at any time prior to the index date within CPRD and THIN. General practitioners in the UK do not have a financial incentive to record pre-existing diagnoses at each visit and only utilizing diagnoses recorded in the 180 days prior to the index date could lead to incomplete comorbidity ascertainment within the UK data sources. Within all data sources, pre-existing microvascular and macrovascular T2DM complications were determined based on diagnoses and surgical procedures recorded within 180 days prior to the index date and categorized according to the Diabetes Complications Severity Index [29].

Within the UK data sources, we collected the closest hemoglobin A1c result recorded in the 180 days prior to the index date. Smoking history and obesity, defined as body mass index (calculated as height in meters/[body weight in kg]^2^) >30 kg/m^2^, were also extracted from CPRD and THIN. Within the US data sources, we collected the number of claims for hemoglobin A1c tests recorded in the 180 days prior to the index date, since patients with unmanaged and/or severe T2DM typically have hemoglobin A1c measured more frequently [30].

Across all data sources, patients were considered exposed to a particular drug if a prescription or pharmacy claim for that drug was recorded within 180 days prior to the index date. Particularly, prior OAD use within the 180 days preceding the index date was determined. Patients whose prescriptions or claims for their existing OAD therapy continued for 90 days before and after the initiation of their index drug were considered to have “added on” saxagliptin or the comparator OAD to their current therapy. Patients whose prescriptions or claims for their existing OAD therapy were recorded 90 days before, but not after, the initiation of the index drug were considered to have “switched to” saxagliptin or a comparator OAD.

### Statistical analysis

Baseline characteristics of new initiators of saxagliptin or other OADs were compared using standardized differences, of which a value exceeding 0.1 is generally considered meaningful [31]. For the purpose of these analyses, standardized difference was calculated as the difference in mean (or proportion for binary variables) divided by the standard deviation (pooled standard deviation for the continuous variables). Conditional logistic regression was used to determine adjusted odds ratios with 95% confidence intervals of saxagliptin use associated with demographic variables, comorbidities, and drug therapies. To explore whether the determinants of saxagliptin use were different between first-time OAD initiators and those who were previously treated with OADs, we re-ran analyses in each database, stratified by whether patients received prior OAD therapy. Because the conditional logistic regression is conditioned on the matched group, initiators who no longer had a matched comparator in the stratified cohorts were removed from this analysis. Data were analyzed using SAS 9.4 (SAS Institute Inc., Cary, NC).

## Results

### Patient characteristics

#### UK data sources

**Figure 1:**
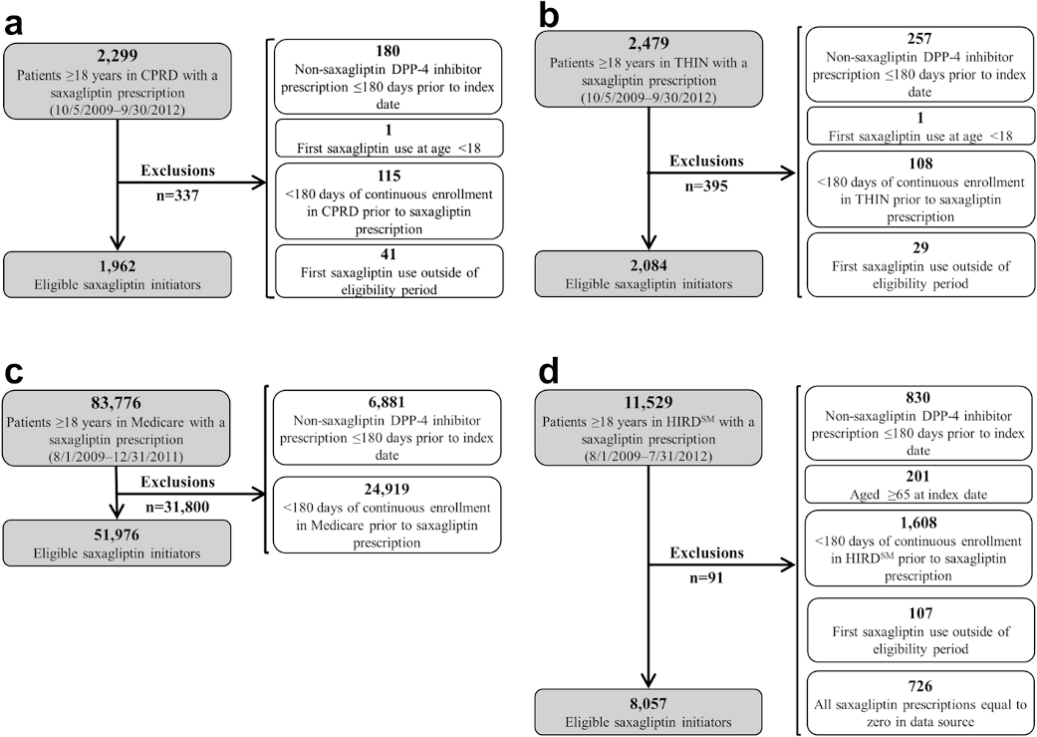
**Selection of saxagliptin patients. a**: Clinical Practice Research Datalink (CPRD). **b**: The Health Improvement Network (THIN). **c**: US Medicare. **d**: HealthCore Integrated Research Database^SM^ (HIRD^SM^).

Within THIN and CPRD, respectively, we identified, 1,962 and 2,084 new initiators of saxagliptin (Figure 1a and b) as well as 19,484 and 19,936 matched new initiators of non-DPP-4 inhibitor OADs. The characteristics of these patients are presented in Table 1. Approximately 6% of saxagliptin initiators in each UK data source had not received treatment for T2DM with another OAD within 180 days prior to the index date.

Within both UK data sources, saxagliptin initiators were more likely to have had prior OAD therapy and hemoglobin A1c results >8% in the 180 days preceding the index prescription compared to initiators of other non-DPP-4 inhibitor OADs. Saxagliptin users also more frequently had hyperlipidemia, hypertension, and obesity and were more commonly prescribed aspirin, anti-hyperlipidemic agents, and anti-hypertensive drugs. Within THIN, diagnoses of retinopathy were more prevalent among saxagliptin initiators.

#### US data sources

During the initial 29 months of saxagliptin availability within US Medicare, 51,976 new initiators of saxagliptin and 493,432 matched new initiators of non-DPP-4 inhibitor OADs were identified (Figure 1c). During the initial 36 months of saxagliptin availability within the HIRD^SM^, 8,057 new initiators of saxagliptin and 77,808 matched new initiators of non-DPP-4 inhibitor OADs were identified (Figure 1d). The characteristics of these patients are presented in Table 2. Approximately 22% of saxagliptin initiators in US Medicare and 33% of saxagliptin initiators in the HIRD^SM^ had not received treatment for T2DM with another OAD within 180 days prior to the index date.

**Table 2:** **Demographic characteristics of type 2 diabetes mellitus patients within United States data sources**

	US Medicare			HealthCore Integrated Reseach Database^SM^		
Characteristic^*^	Saxagliptin	Other OAD	Standardized difference	Saxagliptin	Other OAD	Standardized difference
	(n = 51,976)	(n = 493,432)		(n =8,057)	(n =77,808)	
**Mean (SD) age, years** ^†^	70.4 (11.1)	69.9 (11.0)	0.04	52.9 (8.4)	52.9 (8.4)	0.01
**Male sex** ^†^	42.1%	42.8%	0.01	60.0%	59.5%	0.01
**US census region** ^†^						
East North Central	12.7%	12.8%	<0.01	20.7%	21.2%	0.01
East South Central	10.0%	10.0%	<0.01	10.0%	10.1%	<0.01
Middle Atlantic	16.0%	15.9%	<0.01	9.0%	8.9%	<0.01
Mountain	3.1%	3.1%	<0.01	1.9%	1.9%	<0.01
New England	2.8%	2.8%	<0.01	5.7%	5.9%	<0.01
Pacific	12.8%	13.1%	<0.01	14.6%	14.8%	<0.01
South Atlantic	24.1%	24.0%	<0.01	30.5%	29.9%	0.01
West North Central	5.2%	5.0%	<0.01	5.3%	5.4%	<0.01
West South Central	13.2%	13.2%	<0.01	2.2%	1.9%	0.02
**Other OAD initiated at index date**						
Alpha-glucosidase inhibitors	0%	0.6%	-	0%	0.4%	-
Acarbose	0%	0.5%	-	0%	0.3%	-
Miglitol	0%	0.0%	-	0%	0.0%	-
Biguanide: Metformin	0%	51.5%	-	0%	69.1%	-
Meglitinides	0%	2.4%	-	0%	0.9%	-
Nateglinide	0%	1.0%	-	0%	0.4%	-
Repaglinide	0%	1.3%	-	0%	0.5%	-
Sulfonylureas	0%	33.8%	-	0%	22.0%	-
Chlorpropamide	0%	0.0%	-	0%	0.0%	-
Glimepiride	0%	11.0%	-	0%	8.1%	-
Glipizide	0%	14.3%	-	0%	8.3%	-
Glyburide (glibenclamide in UK data sources)	0%	8.5%	-	0%	5.6%	-
Tolazamide	0%	0.0%	-	0%	0.0%	-
Tolbutamide	0%	0.0%	-	0%	0.0%	-
Thiazolidinediones	0%	11.7%	-	0%	7.7%	-
Pioglitazone	0%	11.0%	-	0%	7.3%	-
Rosiglitazone	0%	0.8%	-	0%	0.4%	-
**On glucagon-like peptide-1 receptor agonist**	2.1%	1.1%	0.07	3.8%	2.9%	0.05
**On insulin**	15.9%	14.9%	0.03	9.7%	8.3%	0.05
**Mean (SD) number of hemoglobin A1c measures**	1.3 (0.9)	0.9 (0.9)	0.51	1.1 (0.8)	0.8 (0.8)	0.50
**Severity of type 2 diabetes mellitus (prior 180 d)**						
Cerebrovascular disease	10.5%	10.6%	<0.01	2.4%	2.4%	<0.01
Coronary artery disease, congestive heart failure, ventricular tachycardia/fibrillation	40.8%	38.2%	0.05	11.8%	11.1%	0.02
Metabolic (ketoacidosis, hyperosmolar,coma)	1.3%	1.3%	<0.01	0.7%	0.7%	<0.01
Nephropathy	20.2%	15.7%	0.11	5.0%	3.6%	0.07
Neuropathy	22.9%	18.1%	0.12	8.3%	6.8%	0.06
Peripheral vascular disease	18.4%	16.6%	0.05	3.9%	3.4%	0.03
Retinopathy	13.3%	10.5%	0.09	5.3%	3.9%	0.07
Unspecified additional diabetic complications	7.9%	6.9%	0.04	3.8%	2.7%	0.06
**Medical comorbidities**						
Allergic rhinitis/hay fever	7.1%	5.6%	0.06	5.2%	4.6%	0.03
Asthma	7.7%	7.9%	0.01	3.8%	4.3%	0.02
Chronic obstructive pulmonary disease/bronchitis	12.5%	13.4%	0.03	2.7%	3.0%	0.02
Dermatologic disorders						
Eczema	3.5%	3.0%	0.02	2.2%	1.9%	0.02
Psoriasis/psoriatic arthritis	0.9%	0.9%	<0.01	0.9%	0.9%	0.01
Gastrointestinal disease						
Cirrhosis	0.8%	0.8%	<0.01	0.3%	0.4%	0.01
Gallbladder disease	2.2%	2.3%	0.01	1.1%	1.1%	<0.01
Hemochromatosis	0.3%	0.2%	0.01	0.2%	0.2%	<0.01
Hyperlipidemia	77.1%	66.3%	0.24	62.0%	48.7%	0.27
Hypertension	85.3%	78.1%	0.18	60.9%	50.9%	0.20
Infections						
Hepatitis B virus infection	0.2%	0.2%	<0.01	0.2%	0.2%	0.01
Hepatitis C virus infection	0.8%	0.9%	0.02	0.5%	0.5%	<0.01
Human immunodeficiency virus	0.2%	0.4%	0.02	0.1%	0.2%	0.03
Malignancy						
Hematologic	1.3%	1.3%	0.01	0.6%	0.6%	0.01
Solid organ	8.2%	8.6%	0.01	3.3%	3.3%	<0.01
Obesity	11.1%	10.7%	0.01	9.3%	9.4%	<0.01
Rheumatoid arthritis	2.8%	2.5%	0.02	0.9%	0.9%	<0.01
**Medications**						
Acetaminophen/paracetamol	28.1%	26.3%	0.04	20.2%	19.8%	0.01
Anti-asthmatic agents	14.4%	12.5%	0.05	8.3%	7.9%	0.01
Antibacterials	46.3%	40.3%	0.12	38.1%	35.7%	0.05
Anticonvulsants	5.4%	5.6%	0.01	7.2%	7.5%	0.01
Antifungals	10.1%	8.1%	0.07	6.8%	5.7%	0.05
Antihistamines	11.8%	9.4%	0.08	6.4%	5.7%	0.03
Anti-hyperlipidemic agents	69.2%	52.8%	0.34	53.5%	38.6%)	0.30
Antihypertensive agents						
Angiotensin-converting enzyme inhibitors	43.1%	37.2%	0.12	35.8%	28.0%	0.17
Angiotensin receptor blockers	28.5%	18.0%	0.25	20.1%	14.1%	0.16
Beta blockers	44.0%	36.1%	0.16	21.2%	18.4%	0.07
Calcium channel blockers	31.7%	26.3%	0.12	16.0%	13.9%	0.06
Loop diuretics	23.1%	18.1%	0.12	5.1%	4.7%	0.02
Other antihypertensive agents	11.9%	9.0%	0.10	4.8%	3.9%	0.04
Thiazide diuretics	21.7%	15.6%	0.16	18.2%	15.0%	0.09
Antivirals	2.3%	2.0%	0.02	1.9%	2.5%	0.04
Non-aspirin non-steroidal anti-inflammatory	17.4%	14.7%	0.08	13.9%	12.9%	0.03
Other antiplatelet/anticoagulant agents						
Aspirin	0.8%	0.6%	0.02	0.1%	0.1%	0.01
Clopidogrel	13.7%	10.4%	0.10	4.0%	3.6%	0.02
Low-molecular-weight heparin	0.5%	0.7%	0.02	0.4%	0.4%	0.01
Warfarin	7.4%	7.0%	0.02	2.1%	1.7%	0.03
Other medications						
Allopurinol	5.4%	4.1%	0.06	2.5%	2.4%	<0.01
Anti-arrhythmics	13.6%	12.2%	0.04	4.7%	4.6%	0.01
Immune modulators/immunosuppressants	4.5%	4.1%	0.02	2.2%	2.4%	0.01
Nitroglycerin	4.6%	3.8%	0.04	1.3%	1.3%	<0.01
Urinary anti-spasmodics	5.4%	4.5%	0.04	0.9%	1.2%	0.03
Psychotropic agents						
Antidepressants	27.4%	25.3%	0.05	19.1%	18.9%	<0.01
Antipsychotics	7.5%	7.8%	0.01	2.1%	2.3%	0.01
**Prior OAD Therapy** ^‡^	77.6%	36.1%	0.92	66.7%	23.2%	0.97
Alpha-glucosidase inhibitors	0.8%	0.3%	0.08	0.4%	0.1%	0.05
Acarbose	0.7%	0.2%	0.07	0.4%	0.1%	0.05
Miglitol	0.1%	0.0%	0.03	0.0%	0.0%	0.02
Biguanide: Metformin	51.4%	37.4%	0.29	51.9%	42.6%	0.19
Meglitinides	3.0%	1.1%	0.13	1.4%	0.4%	0.10
Nateglinide	1.5%	0.5%	0.10	0.8%	0.2%	0.08
Repaglinide	1.6%	0.7%	0.09	0.7%	0.2%	0.07
Sulfonylureas	42.5%	16.8%	0.59	28.0%	8.7%	0.51
Chlorpropamide	0.0%	0.0%	<0.01	0.0%	0.0%	0.01
Glimepiride	16.5%	5.3%	0.37	12.3%	3.2%	0.34
Glipizide	17.7%	8.8%	0.27	11.0%	4.3%	0.26
Glyburide	9.5%	5.2%	0.16	5.2%	2.0%	0.17
Tolazamide	0.0%	0.0%	0.01	0%	0%	-
Tolbutamide	0.0%	0.0%	<0.01	0%	0%	-
Thiazolidinediones	24.2%	8.4%	0.44	15.0%	5.5%	0.32
Pioglitazone	21.4%	7.3%	0.41	13.6%	5.0%	0.30
Rosiglitazone	3.2%	1.9%	0.08	1.5%	0.9%	0.05

Abbreviations: OAD = oral anti-diabetic drug; SD = standard deviation.

^*^Characteristics are presented as percentages unless otherwise indicated.

^†^Matching criteria for which a random sample (without replacement) of up to ten new initiators of non-DPP-4 inhibitor OADs were selected for each saxagliptin initiator.

^‡^Defined as use of an oral anti-diabetic drug within the 180 days prior to the initiation of the index drug. Denominator adjusted to exclude those on index drug.

Within both US databases, saxagliptin initiators more frequently received prior OAD therapy and had higher mean numbers of hemoglobin A1c measurements in the 180 days prior to the index date. Saxagliptin initiators were also more frequently diagnosed with hyperlipidemia and hypertension and more commonly received anti-hyperlipidemic drugs, angiotensin-converting enzyme inhibitors, and angiotensin receptor blockers. Within US Medicare, diagnoses of microvascular T2DM complications, including nephropathy and neuropathy, were more prevalent among saxagliptin initiators.

### Factors associated with saxagliptin use

#### UK data sources

Factors associated with saxagliptin initiation within CPRD and THIN are presented in Table 3. Prior OAD use in the 180 days preceding the index date was strongly associated with saxagliptin initiation in CPRD and THIN. Within THIN, diabetic nephropathy and obesity were also associated with a higher likelihood of saxagliptin initiation.

**Table 3:** **Determinants of saxagliptin use among type 2 diabetes mellitus patients within United Kingdom data sources**

	Adjusted odds ratio (95% confidence interval)^*^					
Characteristic	Clinical Practice Research Datalink			The Health Improvement Network		
	Overall	Prior OAD use	No Prior OAD use	Overall	Prior OAD use	No Prior OAD use
	(n = 21,446)	(n = 8,332)	(n = 890)	(n = 22,020)	(n = 8,621)	(n = 1,137)
Hemoglobin A1c >8%	1.26 (1.13-1.39)	1.21 (1.08-1.35)	1.66 (1.05-2.63)	1.17 (1.06-1.30)	1.12 (1.01-1.25)	1.39 (0.95-2.03)
Overweight vs. < 25 kg/m^2^	0.91 (0.78-1.07)	0.87 (0.73-1.03)	1.32 (0.67-2.62)	1.38 (1.15-1.67)	1.44 (1.17-1.76)	0.95 (0.48-1.87)
Obese vs. < 25 kg/m^2^	1.13 (0.99-1.29)	1.12 (0.97-1.30)	1.61 (0.88-2.94)	1.74 (1.45-2.09)	1.83 (1.50-2.22)	0.86 (0.44-1.68)
Smoking	0.93 (0.81-1.06)	0.94 (0.81-1.08)	0.73 (0.43-1.26)	1.03 (0.93-1.15)	1.03 (0.92-1.16)	0.86 (0.57-1.30)
Severity of type 2 diabetes mellitus						
Cardiovascular	1.05 (0.90-1.23)	1.03 (0.87-1.22)	1.49 (0.74-3.00)	0.97 (0.84-1.13)	1.01 (0.86-1.18)	0.74 (0.43-1.28)
Cerebrovascular	0.79 (0.64-0.98)	0.80 (0.64-1.01)	0.52 (0.20-1.38)	0.87 (0.72-1.05)	0.89 (0.72-1.09)	0.65 (0.31-1.40)
Nephropathy	1.08 (0.78-1.50)	1.05 (0.74-1.50)	1.40 (0.41-4.80)	1.75 (1.33-2.30)	1.77 (1.32-2.39)	1.79 (0.61-5.23)
Peripheral vascular disease	0.88 (0.73-1.07)	0.90 (0.74-1.10)	0.54 (0.21-1.35)	0.83 (0.69-1.00)	0.83 (0.68-1.01)	1.32 (0.65-2.68)
Retinopathy	1.11 (0.98-1.25)	1.06 (0.93-1.20)	2.25 (1.27-3.99)	1.20 (1.06-1.35)	1.14 (1.00-1.29)	2.63 (1.64-4.22)
**Diagnoses**						
Allergic rhinitis/hay fever	0.83 (0.69-1.00)	0.88 (0.73-1.07)	0.36 (0.13-0.97)	0.92 (0.77-1.11)	0.99 (0.82-1.21)	0.52 (0.25-1.07)
Asthma	0.99 (0.83-1.17)	0.96 (0.80-1.15)	1.35 (0.63-2.86)	0.99 (0.84-1.17)	0.96 (0.80-1.15)	1.44 (0.79-2.64)
Chronic obstructive pulmonary disease	1.23 (1.03-1.46)	1.18 (0.98-1.42)	2.64 (1.34-5.18)	1.21 (1.03-1.43)	1.21 (1.01-1.44)	1.48 (0.79-2.77)
Collagen vascular disease/autoimmune disorders	0.96 (0.82-1.13)	0.94 (0.79-1.11)	1.40 (0.64-3.07)	1.01 (0.86-1.17)	1.03 (0.88-1.22)	1.11 (0.59-2.12)
Dermatologic disorders	1.08 (0.96-1.22)	1.12 (0.99-1.27)	0.50 (0.28-0.89)	1.10 (0.98-1.24)	1.14 (1.00-1.29)	0.97 (0.62-1.52)
Hyperlipidemia	1.19 (1.03-1.37)	1.14 (0.98-1.33)	2.08 (1.14-3.81)	1.21 (1.05-1.39)	1.18 (1.02-1.37)	1.04 (0.59-1.83)
Hypertension	0.86 (0.75-0.98)	0.83 (0.73-0.96)	1.14 (0.67-1.93)	0.88 (0.78-1.00)	0.86 (0.75-0.99)	1.39 (0.87-2.22)
Infectious diseases	1.16 (1.01-1.33)	1.14 (0.99-1.32)	1.63 (0.97-2.76)	1.10 (0.96-1.25)	1.08 (0.94-1.24)	1.30 (0.79-2.13)
Malignancy	0.98 (0.87-1.11)	0.95 (0.84-1.08)	1.78 (1.08-2.95)	0.95 (0.85-1.07)	0.96 (0.84-1.09)	0.83 (0.53-1.29)
Obesity	1.07 (0.93-1.23)	0.99 (0.85-1.15)	1.47 (0.83-2.60)	1.00 (0.87-1.16)	0.96 (0.83-1.13)	1.76 (1.03-3.02)
Other diseases	1.08 (0.94-1.25)	1.08 (0.93-1.26)	0.77 (0.40-1.46)	1.12 (0.97-1.28)	1.06 (0.92-1.23)	1.31 (0.78-2.19)
**Drugs**						
Acetaminophen/paracetamol	0.95 (0.84-1.07)	0.96 (0.84-1.09)	0.80 (0.44-1.46)	0.92 (0.82-1.03)	0.88 (0.78-1.00)	1.18 (0.74-1.87)
Anti-asthmatic agents	0.96 (0.81-1.13)	1.03 (0.86-1.23)	0.50 (0.23-1.07)	0.92 (0.79-1.09)	0.96 (0.81-1.14)	0.78 (0.43-1.44)
Antibacterial agents	1.03 (0.92-1.16)	1.00 (0.89-1.13)	1.36 (0.82-2.26)	0.99 (0.88-1.11)	0.98 (0.87-1.10)	1.08 (0.69-1.69)
Anticonvulsants	1.16 (0.96-1.40)	1.19 (0.98-1.46)	0.87 (0.32-2.37)	1.07 (0.86-1.33)	1.05 (0.83-1.33)	0.95 (0.39-2.35)
Antihistamines	0.93 (0.76-1.14)	0.91 (0.73-1.12)	1.57 (0.61-4.04)	1.01 (0.83-1.23)	0.98 (0.79-1.22)	1.35 (0.65-2.77)
Antihyperlipidemic agents	1.33 (1.16-1.52)	1.21 (1.04-1.39)	2.79 (1.60-4.85)	1.32 (1.15-1.51)	1.19 (1.03-1.38)	2.41 (1.50-3.88)
Antihypertensive agents						
Angiotensin-converting enzyme inhibitors	1.11 (0.98-1.26)	1.12 (0.99-1.28)	0.80 (0.44-1.47)	1.00 (0.88-1.12)	0.98 (0.86-1.11)	0.88 (0.55-1.41)
Angiotensin receptor blockers	1.35 (1.16-1.57)	1.36 (1.16-1.60)	1.34 (0.65-2.75)	1.32 (1.14-1.52)	1.29 (1.11-1.51)	1.46 (0.83-2.58)
Beta blockers	1.04 (0.90-1.19)	1.03 (0.89-1.18)	1.46 (0.78-2.72)	1.09 (0.96-1.24)	1.05 (0.92-1.21)	1.49 (0.90-2.45)
Calcium channel blockers	1.00 (0.88-1.13)	1.04 (0.91-1.19)	0.29 (0.15-0.58)	0.94 (0.84-1.06)	0.99 (0.88-1.13)	0.51 (0.31-0.86)
Loop diuretics	0.95 (0.80-1.12)	0.91 (0.76-1.09)	1.00 (0.46-2.19)	1.40 (1.20-1.63)	1.31 (1.11-1.55)	1.94 (1.07-3.52)
Other antihypertensive agents	1.12 (0.97-1.29)	0.98 (0.81-1.19)	2.03 (0.82-5.03)	1.18 (1.02-1.36)	0.91 (0.74-1.12)	1.73 (0.76-3.95)
Thiazide diuretics	1.01 (0.84-1.22)	1.18 (1.02-1.37)	0.39 (0.17-0.94)	0.97 (0.80-1.18)	1.27 (1.10-1.48)	0.54 (0.28-1.02)
Anti-infective agents	0.97 (0.74-1.28)	1.00 (0.75-1.34)	0.59 (0.13-2.64)	0.78 (0.60-1.02)	0.72 (0.54-0.95)	0.80 (0.29-2.26)
Antiplatelet/anticoagulant agents	0.91 (0.81-1.03)	0.90 (0.79-1.02)	1.16 (0.65-2.07)	1.05 (0.93-1.18)	1.01 (0.89-1.14)	1.53 (0.94-2.50)
Non-steroidal anti-inflammatory agents	0.98 (0.84-1.14)	0.90 (0.79-1.02)	0.71 (0.30-1.69)	0.98 (0.84-1.15)	1.05 (0.89-1.24)	0.48 (0.24-0.96)
Other medications	1.03 (0.89-1.19)	1.03 (0.89-1.20)	0.63 (0.31-1.29)	1.09 (0.95-1.25)	1.12 (0.97-1.30)	0.79 (0.44-1.41)
Psychotropic agents	0.94 (0.83-1.07)	0.99 (0.87-1.13)	0.51 (0.25-1.05)	0.95 (0.83-1.07)	0.97 (0.85-1.11)	0.58 (0.34-1.00)

*Abbreviations*: OAD = oral anti-diabetic drug.

^*^Odds ratios adjusted for all other variables in this table.

After stratifying on prior OAD use within CPRD, results were similar to those in the primary analysis. However, prescriptions for antihyperlipidemic agents and diagnoses of chronic obstructive pulmonary disease and bronchitis were associated with saxagliptin initiation among patients without prior OAD use (Table 3).

After stratifying on prior OAD use within THIN, results were also similar to those in the primary analysis. However, obesity and diabetic nephropathy were more strongly associated with saxagliptin initiation among those with prior OAD use. Additionally, among patients with no prior OAD use, prescriptions for antihyperlipidemics and diagnoses of retinopathy were associated with saxagliptin initiation (Table 3).

#### US data sources

Factors associated with saxagliptin initiation in US Medicare and the HIRD^SM^ are presented in Table 4. Saxagliptin initiation was associated with prior OAD use and a greater number of hemoglobin A1c measurements in the 180 days preceding the index date in Medicare and the HIRD^SM^. Additionally, within Medicare, saxagliptin initiators were more likely to receive angiotensin-receptor blockers.

**Table 4:** **Determinants of saxagliptin use among type 2 diabetes mellitus patients within United States data sources**

	Adjusted odds ratio (95% confidence interval)^*^					
Characteristic	US Medicare			HealthCore Integrated Research Database^SM^		
	Overall	Prior OAD use	No prior OAD use	Overall	Prior OAD use	No prior OAD use
	(n = 545,408)	(n = 177,791)	(n = 82,840)	(n = 85,865)	(n = 17,177)	(n = 22,803)
Number of hemoglobin A1c measurements						
1 vs. 0 measured	2.10 (2.04-2.16)	2.09 (2.02-2.16)	2.02 (1.91-2.12)	1.67 (1.56-1.78)	1.40 (1.27-1.54)	1.96 (1.77-2.17)
2+ vs. 0 measured	2.93 (2.85-3.01)	2.87 (2.77-2.97)	2.95 (2.78-3.12)	2.33 (2.16-2.51)	1.91 (1.72-2.12)	1.96 (1.77-2.17)
Severity of type 2 diabetes mellitus						
Cardiovascular	0.95 (0.93-0.97)	0.95 (0.93-0.98)	0.95 (0.90-1.00)	0.96 (0.88-1.05)	0.97 (0.86-1.10)	1.00 (0.76-1.32)
Cerebrovascular	0.90 (0.88-0.93)	0.92 (0.88-0.95)	0.87 (0.82-0.94)	0.92 (0.78-1.09)	0.93 (0.74-1.17)	0.90 (0.77-1.06)
Metabolic (ketoacidosis, hyperosmolar, coma)	0.95 (0.87-1.03)	0.91 (0.82-1.01)	1.06 (0.89-1.27)	-	-	-
Nephropathy	1.12 (1.09-1.15)	1.08 (1.05-1.12)	1.25 (1.18-1.32)	1.04 (0.93-1.18)	0.87 (0.75-1.02)	1.40 (1.14-1.72)
Neuropathy	1.11 (1.08-1.14)	1.08 (1.05-1.11)	1.22 (1.15-1.28)	1.01 (0.92-1.11)	1.05 (0.92-1.19)	1.03 (0.87-1.22)
Peripheral vascular disease	0.97 (0.94-1.00)	0.97 (0.94-1.01)	0.96 (0.91-1.02)	1.06 (0.92-1.21)	0.92 (0.77-1.11)	1.30 (1.03-1.63)
Retinopathy	1.03 (1.00-1.06)	1.01 (0.97-1.04)	1.09 (1.02-1.16)	1.02 (0.91-1.14)	0.95 (0.82-1.10)	1.11 (0.90-1.37)
Unspecified additional diabetic complications^†^	0.96 (0.92-0.99)	0.91 (0.82-1.01)	1.12 (1.04-1.22)	1.01 (0.89-1.14)	0.98 (0.83-1.16)	1.17 (0.93-1.47)
**Diagnoses**						
Allergic rhinitis/hay fever	1.16 (1.12-1.21)	1.19 (1.13-1.25)	1.14 (1.05-1.23)	1.12 (1.00-1.26)	1.29 (1.09-1.52)	0.95 (0.78-1.15)
Asthma	0.91 (0.87-0.94)	0.89 (0.85-0.94)	0.94 (0.87-1.02)	0.83 (0.72-0.95)	0.92 (0.76-1.13)	0.80 (0.63-1.02)
Chronic obstructive pulmonary disease	0.92 (0.89-0.95)	0.92 (0.88-0.95)	0.89 (0.83-0.95)	0.93 (0.79-1.08)	0.88 (0.70-1.09)	0.93 (0.72-1.21)
Collagen vascular disease/ autoimmune disorders	1.01 (0.95-1.08)	1.05 (0.97-1.14)	0.93 (0.82-1.06)	0.90 (0.79-1.03)	0.94 (0.79-1.12)	0.82 (0.66-1.03)
Rheumatoid arthritis^‡^	1.10 (1.03-1.16)	1.08 (1.00-1.17)	1.17 (1.04-1.32)	-	-	-
Spondyloarthritis^‡^	1.04 (1.00-1.09)	1.05 (1.00-1.10)	1.03 (0.95-1.12)	-	-	-
Dermatologic disorders	1.04 (0.99-1.10)	1.02 (0.96-1.09)	1.08 (0.98-1.20)	1.07 (0.93-1.23)	1.10 (0.90-1.34)	1.14 (0.90-1.44)
Psoriasis^‡^	0.97 (0.88-1.08)	0.99 (0.87-1.12)	0.86 (0.70-1.06)	-	-	-
Hyperlipidemia	1.11 (1.08-1.14)	1.11 (1.08-1.15)	1.11 (1.05-1.17)	1.17 (1.10-1.24)	1.17 (1.08-1.27)	1.11 (1.01-1.22)
Hypertension	1.00 (0.97-1.03)	1.01 (0.97-1.04)	0.99 (0.93-1.05)	1.13 (1.07-1.20)	1.17 (1.08-1.27)	1.18 (1.07-1.31)
Infectious diseases	0.86 (0.79-0.94)	0.88 (0.79-0.99)	0.85 (0.71-1.02)	0.89 (0.79-1.01)	1.17 (1.08-1.27)	0.95 (0.78-1.16)
Cellulitis^‡^	0.92 (0.88-0.95)	0.91 (0.87-0.95)	0.92 (0.85-1.00)	-	-	-
Malignancy	0.93 (0.90-0.97)	0.95 (0.91-0.99)	0.89 (0.83-0.96)	1.02 (0.90-1.17)	1.17 (1.08-1.27)	1.03 (0.83-1.27)
Obesity	0.94 (0.91-0.97)	0.96 (0.92-0.99)	0.90 (0.84-0.97)	0.88 (0.81-0.96)	0.95 (0.84-1.07)	0.76 (0.65-0.88)
Other diseases	-	-	-	0.92 (0.77-1.09)	0.94 (0.74-1.20)	0.93 (0.69-1.24)
Alcohol diseases^‡^	0.68 (0.61-0.76)	0.71 (0.62-0.81)	0.90 (0.84-0.97)	-	-	-
Gastrointestinal diseases^‡^	1.01 (0.95-1.06)	0.98 (0.91-1.05)	1.07 (0.95-1.20)	-	-	-
Neurological diseases^‡^	0.76 (0.63-0.93)	0.86 (0.68-1.08)	0.53 (0.35-0.82)	-	-	-
**Drugs**						
Acetaminophen/paracetamol	0.97 (0.94-0.99)	0.97 (0.94-1.00)	0.96 (0.91-1.01)	0.97 (0.91-1.04)	1.02 (0.93-1.12)	0.87 (0.78-0.98)
Anti-asthmatic agents	1.08 (1.05-1.12)	1.09 (1.05-1.14)	1.08 (1.01-1.16)	1.02 (0.93-1.13)	1.02 (0.88-1.17)	1.06 (0.89-1.25)
Antibacterial agents	1.08 (1.06-1.10)	1.08 (1.05-1.11)	1.08 (1.03-1.13)	1.07 (1.01-1.13)	1.12 (1.04-1.21)	0.95 (0.87-1.04)
Anticonvulsants	0.89 (0.85-0.93)	0.91 (0.87-0.96)	0.81 (0.74-0.90)	0.96 (0.87-1.06)	1.01 (0.88-1.16)	0.89 (0.75-1.07)
Antihistamines	1.10 (1.07-1.14)	1.10 (1.06-1.15)	1.09 (1.01-1.17)	1.05 (0.94-1.16)	1.05 (0.91-1.22)	1.11 (0.93-1.32)
Antihyperlipidemic agents	1.22 (1.19-1.24)	1.15 (1.12-1.19)	1.35 (1.29-1.41)	1.13 (1.07-1.19)	1.11 (1.03-1.20)	1.16 (1.06-1.28)
Antihypertensive agents						
Angiotensin-converting enzyme inhibitors	0.98 (0.96-1.01)	0.96 (0.94-0.99)	1.00 (0.96-1.05)	0.97 (0.91-1.02)	0.92 (0.85-1.00)	1.01 (0.90-1.12)
Angiotensin receptor blockers	1.39 (1.36-1.43)	1.32 (1.28-1.36)	1.59 (1.51-1.68)	1.22 (1.13-1.31)	1.07 (0.97-1.19)	1.44 (1.26-1.65)
Beta blockers	1.04 (1.02-1.06)	1.05 (1.02-1.08)	1.02 (0.98-1.07)	0.99 (0.93-1.06)	1.10 (1.00-1.20)	0.86 (0.76-0.97)
Calcium channel blockers	1.02 (0.99-1.04)	1.01 (0.98-1.04)	1.02 (0.97-1.07)	0.97 (0.90-1.04)	1.04 (0.94-1.14)	0.86 (0.76-0.99)
Loop diuretics	1.10 (1.07-1.13)	1.09 (1.05-1.12)	1.13 (1.07-1.20)	0.94 (0.83-1.06)	0.92 (0.79-1.08)	0.99 (0.80-1.22)
Other antihypertensive agents	1.11 (1.07-1.14)	1.11 (1.07-1.15)	1.09 (1.02-1.17)	1.06 (0.94-1.20)	1.05 (0.96-1.16)	0.99 (0.79-1.24)
Thiazidediuretics	1.14 (1.11-1.17)	1.14 (1.11-1.18)	1.14 (1.08-1.21)	0.99 (0.93-1.07)	1.05 (0.96-1.16)	0.83 (0.73-0.95)
Anti-infective agents	-	-	-	1.04 (0.95-1.14)	1.00 (0.88-1.14)	1.01 (0.86-1.18)
Antifungals^‡^	1.10 (1.06-1.14)	1.06 (1.02-1.10)	1.21 (1.13-1.30)	-	-	-
Antivirals^‡^	1.09 (1.02-1.16)	1.10 (1.02-1.19)	1.07 (0.93-1.23)	-	-	-
Antiplatelet/anticoagulant agents	0.94 (0.86-1.02)	0.97 (0.87-1.07)	0.91 (0.75-1.10)	0.98 (0.88-1.10)	0.98 (0.84-1.13)	1.07 (0.87-1.32)
Clopidogrel^†^	1.13 (1.10-1.17)	1.12 (1.08-1.16)	1.18 (1.11-1.26)	-	-	-
Warfarin^†^	0.96 (0.92-0.99)	0.99 (0.94-1.04)	0.85 (0.78-0.93)	-	-	-
Non-steroidal anti-inflammatory agents	1.06 (1.03-1.09)	1.05 (1.02-1.09)	1.09 (1.03-1.15)	1.02 (0.95-1.10)	1.05 (0.94-1.16)	0.99 (0.87-1.12)
Other medications	1.06 (1.01-1.12)	1.08 (1.01-1.15)	1.08 (0.96-1.22)	0.88 (0.81-0.96)	0.83 (0.75-0.93)	0.99 (0.85-1.14)
Allopurinol^‡^	1.05 (1.01-1.10)	1.06 (1.00-1.11)	1.07 (0.97-1.18)	-	-	-
Anti-arrhythmics^‡^	0.99 (0.96-1.02)	0.99 (0.95-1.02)	1.00 (0.94-1.08)	-	-	-
Immune modulators/ suppressants^‡^	1.05 (1.00-1.10)	1.01 (0.95-1.07)	1.16 (1.04-1.28)	-	-	-
Nitroglycerin^‡^	0.98 (0.94-1.03)	0.97 (0.92-1.03)	1.01 (0.92-1.13)	-	-	-
Urinary anti-spasmodics^‡^	1.03 (0.98-1.07)	1.03 (0.98-1.09)	1.05 (0.96-1.16)	-	-	-
Psychotropic agents	0.93 (0.91-0.95)	0.92 (0.89-0.94)	0.92 (0.87-0.96)	0.94 (0.88-1.00)	0.95 (0.87-1.04)	0.87 (0.77-0.97)

*Abbreviations*: OAD = oral anti-diabetic drug.

^*^Odds ratios adjusted for all other variables in this table.

^†^For HIRD^SM^, due to low prevalence, metabolic complications are included in the analyses of unspecified additional diabetic complications.

^‡^Medications and diseases were evaluated separately within US Medicare because of the large sample size within this data source.

After stratifying on prior OAD use within US Medicare, results were similar to those in the primary analysis. However, use of angiotensin-receptor blockers was more strongly associated with saxagliptin use among those without prior OAD use (Table 4).

After stratifying on prior OAD use within the HIRD^SM^, results remained similar to those within the primary analyses (Table 4).

## Discussion

Drug utilization studies can reveal how medications are administered in clinical practice, identify determinants of drug use, ensure robust prescribing practices [32], and establish topics for further study of drug effectiveness and safety [14]. This study found that across two UK and two US data sources, prior OAD use, hypertension, and hyperlipidemia were associated with initiation of saxagliptin rather than other OADs. Saxagliptin initiation was also associated with hemoglobin A1c results >8% within the UK data sources, and a greater number of hemoglobin A1c measurements in the US data sources. Interestingly, saxagliptin was the first OAD utilized for approximately 6% of patients within the UK data sources, 22% of patients within US Medicare, and 33% of patients within the HIRD^SM^. Results from US Medicare and THIN suggest that saxagliptin may be a preferred treatment in patients with more severe (advanced) T2DM, as evidenced by increased diagnoses for microvascular complications. According to these findings, patients prescribed saxagliptin had higher prevalence of comorbid conditions, poor glycemic control, inadequate response to prior OAD therapy, or contraindications to OADs in other classes.

Stratifying our analyses on prior OAD use demonstrated that some determinants were more strongly associated with saxagliptin initiation among patients who had not received prior OAD therapy, particularly within the UK data sources. One exception was the finding that within THIN, obesity and diabetic nephropathy were more strongly associated with saxagliptin initiation among those with prior OAD use. However, stratifying on prior OAD use reduced the overall sample sizes within each stratum, particularly for patients without prior OAD use. As a result, these findings should be interpreted with caution.

These findings contribute to a growing body of research evaluating the characteristics of patients prescribed DPP-4 inhibitors. In three studies within the Ingenix (now Optum) administrative claims database [33-35], patients treated with sitagliptin, another DPP-4 inhibitor, were more likely to have medical comorbidities (i.e., cardiovascular disease, chronic kidney disease, hypertension, lipid disorders, and neuropathy) and were more frequently prescribed cardiovascular medications and insulin. In two additional studies within the General Electric Healthcare’s Clinical Data Services electronic medical records database, patients prescribed sitagliptin were older and had a higher prevalence of preexisting comorbid conditions than patients prescribed other OAD therapies [36]. Patients prescribed sitagliptin were also more likely to have baseline microvascular and macrovascular complications of T2DM than patients receiving exenatide [37]. Our results expand understanding of the DPP-4 drug class by providing new data on determinants associated with saxagliptin initiation and including large samples of T2DM patients within the US and UK.

The observation that saxagliptin was prescribed to a large proportion of T2DM patients without prior OAD use in the US data sources (22% within US Medicare; 33% within HIRD^SM^) compared to the UK data sources (6%) is surprising given current guidelines recommending use of metformin as first-line OAD therapy [38]. A recent study utilizing the IMS Health Vector One National and Total Patient Tracker databases, a compilation of large commercial outpatient prescription and patient databases in the US, similarly found that 28% of non-insulin OAD users were not prescribed metformin and that DPP-4 inhibitors were the most commonly prescribed new drug class of agents [39]. The reasons why saxagliptin was more commonly prescribed as initial OAD treatment among T2DM patients within the US data sources remain unclear. Decreased metformin use may be due, in part, to contraindications to the medication (e.g. renal insufficiency, active liver disease) [40-45]. Further studies are needed to evaluate the reasons for this deviation from recommended prescribing practices.

Since baseline characteristics of patients with T2DM have been shown to influence the efficacy of anti-diabetic therapy [46,47], it will be important to evaluate the determinants of saxagliptin use identified in this study as effect modifiers and confounders in future comparative effectiveness and safety studies. Our results also provide valuable information on variables that should be considered for inclusion within propensity score analyses of saxagliptin use for future pharmacoepidemiologic studies evaluating the comparative effectiveness and safety of saxagliptin compared to other OADs [18].

A particular strength of our analysis was the inclusion of data from US Medicare. Prior studies that evaluated the characteristics of OAD initiators within the US [33-35,37], but did not include Medicare coverage, likely underrepresented T2DM patients over the age of 65 and may have incompletely captured claims among patients also co-enrolled in Medicare. By examining initiators of saxagliptin and other non-DPP-4 inhibitor OADs within four data sources (including US Medicare) and across two continents, our analyses ensured adequate capture and representation of elderly T2DM patients.

Our study has several potential limitations. First, we were unable to determine the duration of T2DM due to the use of administrative data (US data sources) and incomplete electronic health data from patients who may have switched practices (UK data sources). Second, actual exposure to saxagliptin and other OADs cannot be confirmed. However, minimal misclassification of medication use is expected since prescribing records within the UK data sources and pharmacy claims within the US data sources were used to determine drug exposure. Additionally, all relevant diagnosis and procedure codes were included and reviewed by clinical and pharmacoepidemiology experts to minimize misclassification of medical comorbidities examined as determinants of saxagliptin use. Third, some potentially important variables, including alcohol and illicit drug use, diet, exercise, family history of diseases, and nonprescription drug use, were not recorded within the data sources. Finally, our results may not be generalizable to all settings. However, our analyses have expanded the populations to which these findings can be generalized by examining results from four different data sources within the US and UK [48], which contain claims and medical records data from both private and public health insurance plans.

## Conclusion

In summary, this study found that saxagliptin initiation was more common in patients with prior complications associated with T2DM, prior OAD use, and diagnoses and receipt of treatment for hyperlipidemia and hypertension. These variables should be considered in future studies evaluating the comparative safety and effectiveness of saxagliptin and other OADs.

## Abbreviations

CPRD: Clinical Practice Research Datalink
DPP-4: Dipeptidyl peptidase-4
FDA: US Food and Drug Administration
HIRD^SM^: HealthCore Integrated Research Database^SM^
OAD: Oral anti-diabetic drug
T2DM: Type 2 diabetes mellitus
THIN: The Health Improvement Network
UK: United Kingdom
US: United States
